# Importance of Intracellular Energy Status on High-Hydrostatic-Pressure Inactivation of *sake* Yeast *Saccharomyces cerevisiae*

**DOI:** 10.3390/foods13050770

**Published:** 2024-03-01

**Authors:** Toru Shigematsu, Taisei Kuwabara, Yuki Asama, Rinta Suzuki, Minami Ikezaki, Kazuki Nomura, Saori Hori, Akinori Iguchi

**Affiliations:** 1 Faculty of Applied Life Sciences, Niigata University of Pharmacy and Medical and Life Sciences, 265-1 Higashijima, Akiha-ku, Niigata 956-8603, Japank_nomura@neptune.kanazawa-it.ac.jp (K.N.); ujiie@nupals.ac.jp (S.H.); a_iguchi@nupals.ac.jp (A.I.); 2 Graduate School of Applied Life Sciences, Niigata University of Pharmacy and Medical and Life Sciences, 265-1 Higashijima, Akiha-ku, Niigata 956-8603, Japan; 3 Department of Applied Bioscience, College of Bioscience and Chemistry, Kanazawa Institute of Technology, 7-1 Ohgigaoka, Nonoichi 921-8501, Japan

**Keywords:** *sake* (Japanese rice wine), high hydrostatic pressure (HHP) processing, pasteurization, *Saccharomyces cerevisiae*, *sake* yeast, pressure-sensitive (piezosensitive) mutant, piezotolerance, intracellular ATP concentration, cultivation conditions

## Abstract

The HHP inactivation behaviors of Niigata *sake* yeast *Saccharomyces cerevisiae* strain S9arg and its aerobic respiratory-deficient mutant strains were investigated after cultivating them in a YPD media containing 2% to 15% glucose, as well as in *moromi* mash, in a laboratory-scale *sake* brewing process. The piezotolerance of strain S9arg, shown after cultivation in a YPD medium containing 2% glucose, decreased to become piezosensitive with increasing glucose concentrations in YPD media. In contrast, the piezosensitivity of a mutant strain UV1, shown after cultivation in the YPD medium containing 2% glucose, decreased to become piezotolerant with increasing glucose concentrations in the YPD medium. The intracellular ATP concentrations were analyzed for an *S. cerevisiae* strain with intact aerobic respiratory ability, as well as for strain UV1. The higher concentration of ATP after cultivation suggested a higher energy status and may be closely related to higher piezotolerance for the yeast strains. The decreased piezotolerance of strain S9arg observed after a laboratory-scale *sake* brewing test may be due to a lower energy status resulting from a high glucose concentration in *moromi* mash during the early period of brewing, as well as a lower aeration efficiency during the brewing process, compared with cultivation in a YPD medium containing 2% glucose.

## 1. Introduction

Rice is a major crop around the world, especially in Asian countries, including Japan. Rice can be processed in many ways for numerous food products, such as *sake* (Japanese rice wine). *Sake* is produced via the multiple parallel fermentation processes, which consists of simultaneous saccharification of rice starch by enzymes derived from *koji* mold (*Aspergillus oryzae*) and ethanol fermentation by *sake* yeast (*Saccharomyces cerevisiae*), and it is subsequently thermally pasteurized before shipment. Recently, the market for Japanese sake exports has been increasing from JPY 10.5 billion in 2013 to JPY 47.5 billion in 2022 [1]. However, even in 2022, approximately only 9% of the total *sake* produced in Japan was exported, resulting in a traceable level of global market share of *sake* out of all alcoholic beverages in the world. The expansion of the international market share of *sake* is expected and is also encouraged by the Japanese government [2].

Unpasteurized draft *sake* has a potentially high market value due to its fresh flavor and fruity taste compared with thermally pasteurized *sake*. However, draft *sake* has a short shelf-life due to the rapid deterioration of its flavors and taste by over-fermentation from *sake* yeast and the activity of remaining enzymes produced by *koji* mold. Thus, non-thermal pasteurization technology for *sake* manufacturing may be important to further expand the global market for *sake* as a rice product.

High hydrostatic pressure (HHP) treatment is a non-thermal technique that can inactivate microorganisms at lower temperatures compared with traditional thermal processing. Applications of HHP processing on foods and/or food materials can extend their shelf life while suppressing the deterioration of their fresh taste and flavors [3,4]. The global market for HHP-processed foods reached approximately USD 9.8 billion in 2015 and is expected to attain a market value of USD 54.77 billion by 2025 [5]. Recently, we developed a sparkling-type draft cloudy *sake*, AWANAMA, using HHP treatment as a non-thermal pasteurization method, which retains the sensory characteristics of draft *sake* and has a longer shelf life [6,7].

It has been demonstrated that HHP inactivation behaviors of microorganisms are affected by numerous extracellular and/or intracellular conditions [8,9,10,11]. In addition to the HHP conditions, which are the pressure levels and HHP treatment time and temperature [12,13,14], the inactivation of microorganisms also depends on extracellular environments, such as the composition of suspension media or foods, pH, and water activity [15]. The type of microorganisms, or their genetic backgrounds, also affects HHP inactivation. The growth phase has also been demonstrated to be an important factor affecting HHP inactivation. Microbial cells in stationary phases were reported to be less susceptible to environmental stresses, including HHP, than exponential-phase cells [15]. However, there is limited knowledge concerning the influence of cultivation conditions, such as the composition of the cultivation media, on inactivating microorganisms with HHP treatment. The HHP inactivation of *sake* yeast strains in the *sake* brewing process has not been reported on either.

Previously, we obtained a pressure-sensitive (piezosensitive) mutant strain cultivated on a conventional YPD medium containing 2% (*w*/*v*) glucose from *S. cerevisiae* by random mutagenesis using ultraviolet irradiation (UV) [16]. Genome analysis revealed that this mutant strain had a deletion of the *COX1* gene that encodes subunit I of cytochrome *c* oxidase in mitochondrial DNA; the strain was deficient in aerobic respiration and/or mitochondrial functions [17]. We also obtained piezosensitive mutant strains, which also have a deletion in the *COX1* gene region in mitochondrial DNA, from a Niigata *sake* yeast strain, S9arg [17]. These findings suggest that deficiencies in aerobic respiration and/or mitochondrial function cause the piezosensitivity of yeast. These mutants were also expected to be applied for HHP-processed *sake* under more moderate HHP conditions, lower pressure levels, and/or shorter treatment periods, which would be economically preferable for HHP processing. However, the mechanism by which the deficiency in aerobic respiration and/or mitochondrial function causes piezosensitivity remains unclear. And the piezotolerance and/or piezosensitivity may be affected by cultivation conditions, including the *sake* brewing process. Thus, in this study, we investigated the effects of cultivation conditions, including the *sake* brewing process, on the HPP inactivation behaviors of the *sake* yeast strains. The intracellular adenosine triphosphate (ATP) was also analyzed to evaluate how the energy states depend on the cultivation conditions.

## 2. Materials and Methods

### 2.1. Yeast Strains

A Niigata *sake* yeast *S. cerevisiae* strain S9arg, derived from an original strain, G9 [18], was provided by the Niigata Prefectural Sake Research Institute (Niigata, Japan) and was used in this study. The piezosensitive mutant strains derived from strain S9arg, strain UV1, and NM1 [17] were also used in this study. *S. cerevisiae* strain MTY3143 (NBRP ID: BY29262), which was generated by Takaine, M. et al. (*his3*Δ1::2×pRS303-PTEF-QUEEN-2m *leu2*Δ0 *lys2*Δ0 *ura3*Δ0) [19], was obtained through the Yeast Genetic Resource Centre Japan (YGRC, http://yeast.nig.ac.jp/yeast/top.xhtml (accessed on 7 February 2024)).

### 2.2. Growth Conditions

The strains were usually grown aerobically on conventional YPD (yeast extract, peptone, dextrose) medium (1% (*w*/*v*) yeast extract, 2% (*w*/*v*) peptone; Becton Dickinson and Co., Franklin Lakes, NJ, USA, and 2% (*w*/*v*) glucose; FUJIFILM Wako Pure Chemical, Osaka, Japan) at 30 °C for 48 h with shaking. Modified YPD media with D(+)-glucose concentrations of 5% (*w*/*v*), 10%, and 15% (*w*/*v*) were also used for cultivation.

Minimal medium (0.67% (*w*/*v*) yeast nitrogen base without amino acids; Becton Dickinson and Co., Franklin Lakes, NJ, USA, 2% D(+)-glucose; FUJIFILM Wako Pure Chemical, Osaka, Japan) was prepared to cultivate strain MTY3143.

*Koji* (malted rice) extract medium was prepared as follows. A volume of 500 mL of water was added to 150 g of dry *koji* (provided by a local *sake* brewery, Kanemasu Sake Co., Shibata, Japan). The mixture was incubated for 6 h at 65 °C to allow saccharification of starch in the rice. After incubation, the mixture was centrifuged at 4300× *g* for 10 min. The supernatant was filtered using filter paper (No. 5B, 90 mm; ADVANTEC TOYO, Tokyo, Japan). The resultant filtrate was collected, autoclaved, and used as a *koji* extract medium.

For growth analysis, the yeast strains were cultivated in 100 µL of medium in a 96-well round-bottom microplate (IWAKI 3870-096; AGC Techno Glass Co., Shizuoka, Japan). Cell growth was monitored via the optical density at 660 nm (OD_660_) every 30 min using an automated incubation reader (mixing speed, 10; bio microplate reader HiTS; Scinics Co., Tokyo, Japan).

In this study, the cell concentration was evaluated based on the OD_660_, colony counting, or dry weight. The dry weight of cells in culture broth was measured as follows: a 20 mL portion of culture broth was centrifuged at 5000× *g* at 4 °C for 5 min. The pellet was resuspended with 5 mL of distilled water. The weight and water content of the cell suspension were measured using a moisture determination balance (FD-620; Kett Electric Laboratory Co., Tokyo, Japan) to calculate the dry weight.

### 2.3. Laboratory-Scale Sake Brewing Test

A laboratory-scale *sake* brewing test was carried out according to our previous report [17], which was conducted by Mr. Iwao Takahashi (a master brewer at Kanemasu Sake Co., Shibata, Japan). Yeast strains were pre-cultivated in 3 mL of YPD medium at 30 °C for 24 h with shaking. After pre-cultivation, a 160 μL aliquot of the culture broth was inoculated into 200 mL of YPD medium containing 10% (*w*/*v*) glucose and cultivated at 15 °C for 48 h without shaking. Then, the culture broth was transferred into a 250 mL NALGENE bottle (Thermo Fisher Scientific, Waltham, MA, USA), and the yeast cells were collected by centrifugation at 3000 rpm (4 °C) for 5 min using a KUBOTA 6200 (KUBOTA, Tokyo, Japan). The viable cells after centrifugation were counted using a staining method with 0.4% trypan blue solution (FUJIFILM Wako Pure Chemical, Osaka, Japan). A volume of 4.8 mL of the cell suspension containing 8.4 ± 0.1 log cells of the strains was used as the fermentation starter (*shubo*). Water *koji* was prepared by adding 50 mL of sterilized water to dry *koji* (malted rice provided by Kanemasu Sake Co., Shibata, Japan) and pre-cultivated at 15 °C for 24 h without shaking. The *shubo* and 10% (*w*/*v*) lactic acid solution were followed by steamed rice, dry *koji*, and sterilized water, which were divided into three portions and added to prepare the fermentation mash (*moromi*). This three-stage traditional mashing method is called *sandan-jikomi*, and the stages of adding materials are called *hatsu-zoe* (first edition), *naka-zoe* (second edition), and *tome-zoe* (third edition). The brewing temperatures of the *moromi* were controlled at 15 °C during the *hatsu-zoe*, 9 °C during the *naka-zoe*, and 7 °C during the *tome-zoe* for 1 day. The day of *tome-zoe* (third addition of materials) was defined as Day 0 of brewing, and the temperature was increased by 1 °C per day, from 7 to 15 °C. After Day 8, the brewing temperature was controlled at 15 °C until the end of the brewing period. The brewing behavior was evaluated via the volume decrement in the *moromi* weight due to CO_2_ output during ethanol fermentation. In this study, the brewing was ended on Day 28.

### 2.4. High Hydrostatic Pressure (HHP) Treatment

HHP treatment was carried out according to our previous report [16]. The cultivated yeast cells were collected and diluted 10-fold with sterilized 0.85% (*w*/*v*) NaCl solution. The cell suspension was vacuum packed in polyethylene bags and soaked in distilled water (pressure medium) in a stainless-steel vessel of an HHP apparatus (WIP, Kobe Steel, Kobe, Japan). The HHP treatment was performed at 200 MPa at ambient temperature (approximately 20 °C).

The HHP inactivation of yeast cells was evaluated using viable cell counts with the colony count method. The cell suspensions after HHP treatment were serially diluted with sterilized 0.85% (*w*/*v*) NaCl solution. To determine the viable cell count, these cell suspensions were plated on a compact dry Nissui YM dry sheet medium culture plate (Nissui Pharmaceutical, Yuuki, Japan) and cultivated at 30 °C for 3 days. The viable cell counts were determined from the number of colonies appearing.

The loss of viability was approximated by means of first-order kinetics with the following equations:

*N* = *N*_0_ *e*^−*k t*^ (1)

ln(*N*/*N*_0_) = −*k t* (2)

where *N* is the viable cell count (cfu/mL) at time *t* (s), *N*_0_ is the viable cell count (cfu/mL) at time 0 (s), and *k* is the inactivation rate constant (s^–1^).

### 2.5. Microscopic Observations and QUEEN Protein Assay

Microscopic observations were performed using an epifluorescence microscope (BX41; Olympus Optical Co., Tokyo, Japan) equipped with mirror units (fluorescence filter cubes) U-WBV2 (Olympus Optical Co., Tokyo, Japan) and U-WIB2 (Olympus Optical Co., Tokyo, Japan) for detecting ATP-bound and ATP-free QUEEN proteins, respectively. The microscopic image was captured with an exposure time of 0.8 s and ISO sensitivity of 400. The fluorescence intensity was analyzed using ImageJ image analysis software (ver. 1.54h) (http://rsb.info.nih.gov/ij/ (accessed on 7 February 2024)).

### 2.6. Analyses of Sugars

The sugars in the culture broth or in *moromi* following the laboratory-scale *sake* brewing process were analyzed using high-performance liquid chromatography with a refractive index detector (HPLC-RID). The assembled HPLC system used in this study consisted of the following: a degasser (DG 1580-53, Jasco, Tokyo, Japan); a low gradient unit (LG 2080-02, Jasco); an HPLC pump (PU 2080 Plus, Jasco); an auto-injector (SIL-20A, Shimadzu, Kyoto, Japan); a system controller (SCL-10APVP, Shimadzu, Kyoto, Japan); and a column oven (CTO-10ACVP, Shimadzu, Kyoto, Japan), equipped with an Inertsil NH2 (5 μm) column (4.6 mm × 250 mm; GL Sciences, Tokyo, Japan) temperature-controlled at 35 °C. Acetonitrile (75% *v*/*v*) in distilled water was used as the mobile phase, and the compounds were eluted isocratically using a flow rate of 1 mL/min. The load volume was 20 μL. A refractive index detector (RID-10A, Shimadzu, Kyoto, Japan) was used to detect sugars. Chromatograms were analyzed using Chromato-PRO software (ver. 5.0) (Run Time Corporation, Tokyo, Japan).

### 2.7. Analyses of Adenosine Triphosphate (ATP), Adenosine Diphosphate (ADP), and Adenosine Monophosphate (AMP) in Yeast Cells

Strain UV1 was cultivated in a YPD medium containing 2% (*w*/*v*) or 15% (*w*/*v*) glucose for 48 h at 30 °C, and it was subjected to metabolite extraction. The intracellular metabolites were extracted from cells as described in our previous report [17]. Briefly, a 5 mL portion of the culture broth was mixed with 7 mL of pre-chilled (−60 °C) 60% (*v*/*v*) methanol in water. The mixture was centrifuged at 5000× *g* for 5 min at −9 °C, and the supernatant was discarded. The pellet was resuspended in 80 µL of 1.25 mM 1,4-piperazinediethanesulfonic acid (PIPES), which was used as the internal standard. To extract the metabolites, 75% (*v*/*v*) ethanol, preheated to 95 °C in an aluminum block bath (model DTU-1C, TAITEC, Koshigaya, Japan), was rapidly added to the resuspended cell pellet. The mixture was immediately vortexed, and the sample was placed in the aluminum block bath at 95 °C for 3 min. After cooling on ice, the extracts were evaporated at 40 °C using a centrifugal evaporator (model CVE-3100, Tokyo Rikakikai, Tokyo, Japan). The dried samples were resuspended in 100 µL of milli-Q water. After centrifugation at 20,400× *g* at 4 °C for 5 min (model MX-301, Tomy Seiko, Tokyo, Japan), the supernatants were used for further analysis.

Analyses of ATP, ADP, and AMP were performed using capillary electrophoresis–electrospray ionization-mass spectrometry (CE–ESI-MS). CE–ESI-MS analyses were performed using a P/ACE MDQ capillary electrophoresis system (SCIEX, Framingham, MA, USA) and a 3200 QTRAP LC/MS/MS system (SCIEX, Framingham, MA, USA). CE separations were performed using an 80-cm length of capillary tubing (PN 338472, SCIEX, Framingham, MA, USA) with an inner diameter (I.D.) of 50 µm and an outer diameter (O.D.) of 375 µm. The electrolyte used for CE separation was 50 mmol/L ammonium acetate solution. Before the injection for each analysis, the capillary was pretreated with running electrolyte for 2 min at 5 psi (approximately 34.5 kPa). Then, the sample was injected at a pressure of 5 psi for 10 s, followed by injection of running electrolyte at 5 psi for 5 s. The voltage applied for separation was +30 kV for 8 min. Voltage application was stopped after 8 min, and the electrolyte was then delivered through the capillary at 4.5 psi (approximately 31.0 kPa) for 13 min. The capillary was maintained at 20 °C throughout the process. Sheath liquid (5 mmol/L ammonium formate in 50% (*v*/*v*) acetonitrile/water) was delivered to the electrospray probe at a rate of 5 µL/min. ESI-MS/MS was conducted in the negative ion mode. Ion spray voltage was applied at −4.5 kV, starting 1 min after the start of voltage application for CE. The metabolites in the samples separated by CE were detected via MS/MS in the multiple reaction monitoring (MRM) mode. The measurement parameters of ESI-MS/MS for each analyzed metabolite were optimized using Analyst software (ver. 1.5) (SCIEX, Framingham, MA, USA); the resultant Q1 (m/z of deprotonated precursor ion) and Q3 (*m*/*z* of the fragmented ion) are as follows: ATP, Q1 505.448 m/z and Q3 158.500 m/z; ADP, Q1 425.493 *m*/*z* and Q3 78.900 *m*/*z*; AMP, Q1 345.594 *m*/*z* and Q3 79.000 *m*/*z*.

## 3. Results and Discussion

### 3.1. HHP Inactivation Behavior of the Strains Cultivated in a YPD Medium Containing 2% Glucose or after a Laboratory-Scale Sake Brewing Test

The HHP inactivation behaviors were analyzed for yeast strain S9arg (wildtype) and its piezosensitive mutants UV1 and NM1, cultivated on conventional YPD medium containing 2% (*w*/*v*) glucose or in a laboratory-scale *sake* brewing test. Strains UV1 and NM1 were obtained by UV mutagenesis. Both mutants showed deletions in *COX1* and *COB* gene regions in mitochondrial DNA [17].

**Figure 1:**
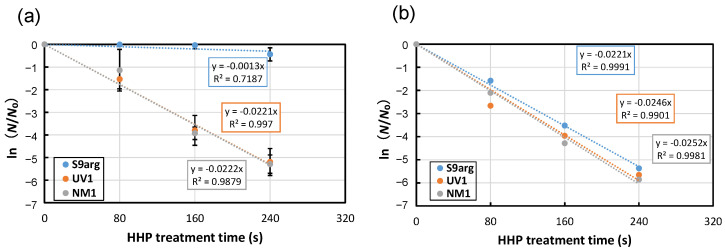
Pressure inactivation curves at 200 MPa at ambient temperature (approximately 20 °C) of yeast strains S9arg (blue circles), UV1 (orange circles), and NM1 (gray circles) after cultivation in YPD medium (2% glucose) for 60 h at 30 °C (**a**), or after 28 d of brewing in a laboratory-scale *sake* brewing test (**b**). Average values from three assays are shown with error bars indicating standard deviations (**a**) or values from a single assay (**b**). Approximate curves were shown in dotted lines with equations.

**Figure 2:**
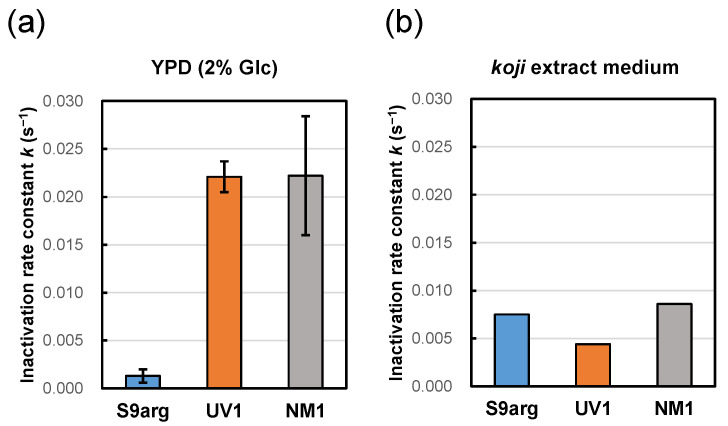
Pressure inactivation rate constants (*k*) at 200 MPa at ambient temperature (approximately 20 °C) of yeast strains S9arg (blue), UV1 (orange), and NM1 (gray) after cultivation in YPD medium containing 2% glucose (**a**), and on *koji* (malted rice) extract medium (**b**) for 48 h at 30 °C. Average values from three assays are shown with error bars indicating standard deviations (**a**) or values from a single assay (**b**).

After 48 h cultivation of YPD medium containing 2% glucose, the cell suspensions of the stationary phase were subjected to HHP treatment at 200 MPa at ambient temperature (approximately 20 °C). Decreased viability was confirmed for all three strains tested, and the inactivation followed first-order kinetics (Figure 1a). From the slopes of approximate curves, the average inactivation rate constants (*k*) of strains S9arg, UV1, and NM1 were 0.0013 s^−1^, 0.0221 s^−1^, and 0.0222 s^−1^, respectively (Figure 1a and Figure 2a). The inactivation constants of strains UV1 and NM1 were larger than that of strain S9arg, indicating the piezosensitivity of the two mutants.

The cells of the three strains after the 28 d brewing period in a laboratory-scale *sake* brewing test were also subjected to HHP treatment at 200 MPa at ambient temperature. In contrast to the cells cultivated in a YPD medium containing 2% glucose, the inactivation behaviors of all three strains showed roughly similar inactivation rate constants of 0.0211 s^−1^ to 0.0252 s^−1^ (Figure 1b). The results indicated increased piezosensitivity of strain S9arg after the laboratory-scale *sake* brewing test. The piezosensitivities of mutant strains UV1 and NM1 were, contrarily, approximately comparable with those of cells after cultivation in a YPD medium containing 2% glucose. These results suggest that the piezotolerance of strain S9arg decreased under the environment of the *sake* brewing process, but the piezotolerance of the UV1 and NM1 mutant strains were not affected under the environment of the *sake* brewing process.

Generally, during the *sake* brewing process, glucose concentration in the early brewing period is high, roughly 10% (*w*/*v*); subsequently, the concentration decreases and reaches low levels below 1%. The glucose concentration in the *moromi* mash after our brewing test was approximately 0.18% (*w*/*v*), regardless of the *sake* yeast strain. In addition to glucose, the *moromi* mash contained numerous complex compounds. Some of them were derived from the *koji* (malted rice).

To mimic the environments of *moromi* mash, a *koji* extract medium was prepared to cultivate the three yeast strains. After 48 h cultivation of strains S9arg, UV1, and NM1 at 30 °C, the cell suspensions were subjected to HHP treatment at 200 MPa at ambient temperature. The pressure inactivation rate constant of strain S9arg was 0.0075 s^−1^, which was larger than that after cultivation in a YPD medium containing 2% glucose. In contrast, the pressure inactivation rate constants of strains UV1 and NM1 were 0.0044 s^−1^ and 0.0086 s^−1^, respectively, which were significantly smaller than those after cultivation in a YPD medium containing 2% glucose (Figure 2). The inactivation behavior differed depending on the cultivation medium. Cultivation in the *koji* extract medium caused increased inactivation of strain S9arg compared with cultivation in the YPD medium, which was similar to cells in the *sake* brewing test. Interestingly, in the mutant strains UV1 and NM1, the cultivation of *koji* extract medium caused decreased inactivation compared with cultivation in YPD medium containing 2% glucose.

The sugars in the YPD and *koji* extract media were analyzed with HPLC-RID. The most apparent difference was in the glucose concentrations, which were approximately 2% (*w*/*v*) in YPD and 14.6% (*w*/*v*) in *koji* extract media, respectively. These results suggest that the initial glucose concentration of the cultivation medium affects the variation in piezotolerance or piezosensitivity of yeast cells after cultivation.

As the results described above, as well as our previous report [13], the two mutant strains UV1 and NM1 showed essentially comparable HHP inactivation behaviors as well as brewing characteristics. Thus, strain UV1 was selected for further study as a strain with deficient mitochondrial functions.

### 3.2. Growth Analysis of Strains S9arg and UV1 in a YPD Media Containing 2% to 15% Glucose

**Figure 3:**
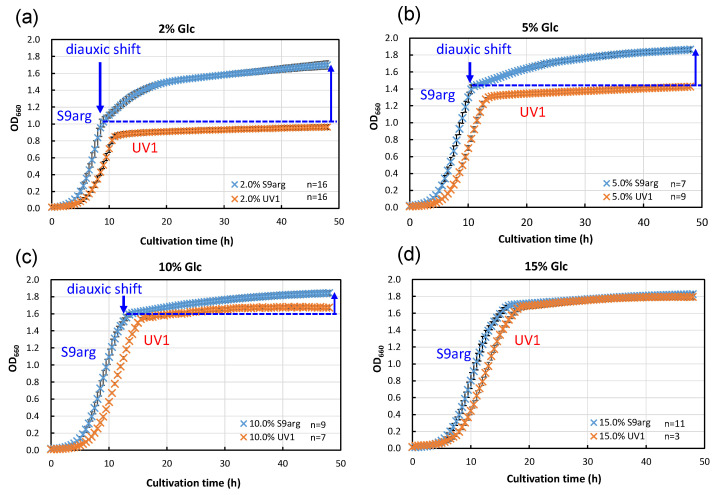
Growth curves of yeast strains S9arg (blue crosses) and UV1 (orange crosses) in a YPD media containing 2% (*w*/*v*) (**a**), 5% (*w*/*v*) (**b**), 10% (*w*/*v*) (**c**), and 15% (*w*/*v*) (**d**) of glucose at 30 °C. Average values are shown with error bars indicating standard deviations. The numbers of experiments are shown on the panels.

We carried out an analysis of the HHP inactivation behaviors of strain S9arg, as well as the mutant strain UV1, cultivated in a YPD media containing different concentrations of glucose. YPD media containing 2% (*w*/*v*), 5% (*w*/*v*), 10% (*w*/*v*), and 15% (*w*/*v*) glucose were prepared. The concentrations of glucose in the YPD media were designed as follows: the lowest 2% reflected the concentration in the YPD medium popularly used for yeast cultivation, and the highest 15% roughly reflected the concentration in the *koji* extract medium (14.6%). Strains S9arg and UV1 were cultivated on YPD and the modified YPD media at 30 °C for 48 h. The cell growth rates were monitored and are shown in Figure 3. When strain S9arg was cultivated in a YPD medium containing 2% glucose, strain S9arg showed exponential growth for 8 h, and then the growth decelerated to a slower secondary growth to 48 h (Figure 3a). The initial glucose in the medium was utilized for ethanol fermentation for 8 h by the Crabtree effect [20]. When the glucose concentration reached a low level, a diauxic shift from ethanol fermentation to aerobic respiration for energy metabolisms occurred since the strain had intact respiratory function. The diauxic shift was also detected in cultivations in a YPD media containing 5% and 10% glucose (Figure 3b,c), whereas a diauxic shift was not detected in a YPD media containing 15% glucose (Figure 3d).

In contrast, strain UV1 cultivated in a YPD medium containing 2% glucose did not show a diauxic shift, resulting in no apparent secondary growth after 10 h to 48 h cultivation (Figure 3a). This strain did not show a diauxic shift, regardless of the glucose concentrations in YPD media. The mutant strain is deficient in aerobic respiration, which is caused by the deletion of *COX1* and *COB* gene regions in mitochondrial DNA [17].

**Figure 4:**
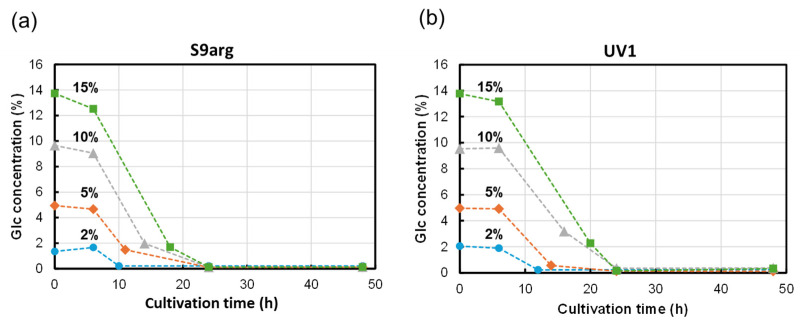
Glucose concentrations in culture broth during cultivation of yeast strains S9arg (**a**) and UV1 (**b**) in a YPD media containing 2% (*w*/*v*) (blue circles), 5% (*w*/*v*) (orange diamonds), 10% (*w*/*v*) (gray triangles), and 15% (*w*/*v*) (green boxes) of glucose at 30 °C.

The glucose in the media was analyzed during cultivation with HPLC-RID, as shown in Figure 4. During the cultivation of strain S9arg, the concentration of glucose drastically decreased after the start of cultivation. Low levels of glucose were detected after 10 h, 12 h, 14 h, and 18 h of cultivation in a YPD media containing 2%, 5%, 10%, and 15% glucose, respectively (Figure 4a). During the cultivation of strain UV1, the decreases in glucose concentrations were approximate to those observed when cultivating strain S9arg. Low levels of glucose were detected after 12 h, 14 h, 24 h, and 24 h of cultivation in a YPD media containing 2%, 5%, 10%, and 15% glucose, respectively (Figure 4b). For both strains, only trace levels of glucose were detected after 24 h of cultivation, regardless of the initial glucose concentrations, suggesting that the glucose in the YPD medium was mostly used up.

### 3.3. HHP Inactivation Behavior of the Strains Cultivated in a YPD Media Containing 2% to 15% Glucose

**Figure 5:**
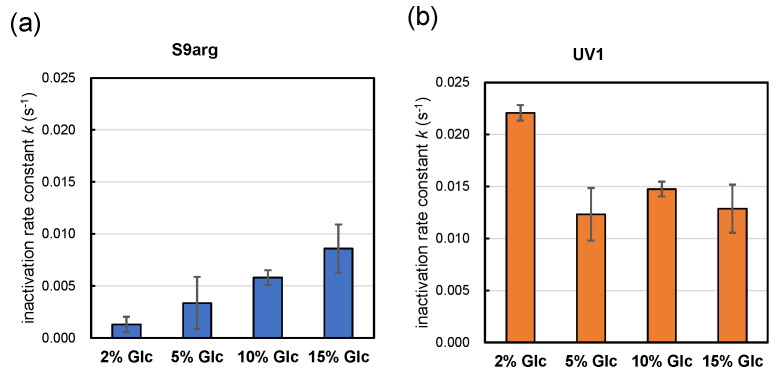
Pressure inactivation rate constants *k* (s^−1^) of yeast strains S9arg (**a**) and UV1 (**b**) in a YPD media containing 2% (*w*/*v*), 5% (*w*/*v*), 10% (*w*/*v*), and 15% (*w*/*v*) of glucose for 48 h at 30 °C. The *k* values represent averages from three assays, with error bars indicating standard deviations, with one exception: the 10% glucose results show averages of two assays.

Strains S9arg and UV1 were cultivated in a YPD media containing 2%, 5%, 10%, and 15% glucose for 48 h. Then, the resultant cells were subjected to HHP treatment at 200 MPa at ambient temperature for 240 s. The calculated inactivation rate constants (*k*) for strain S9arg are shown in Figure 5a and Table 1. The *k* value increased with increases in the initial glucose concentration in the YPD media, being 0.0013 s^−1^ (2% Glc) to 0.0086 s^−1^ (15% Glc). The *k* value of strain S9arg cultivated on *koji* extract medium was 0.0075 s^−1^ (Figure 2b), which was roughly comparable with that cultivated in a YPD medium containing 15% glucose. Thus, the increased piezosensitivity of the strain cultivated on the *koji* extract medium could be explained by the initial glucose concentration used.

In contrast, the *k* value of strain UV1 decreased with increases in the initial glucose concentration in the YPD medium, being 0.0221 s^−1^ (2% Glc) to 0.0129 s^−1^ (15% Glc) (Figure 5b, Table 1). The *k* value of strain UV1 cultivated on *koji* extract medium was 0.0044 s^−1^ (Figure 2b), which was quite different from 0.0129 s^−1^ for UV1 cultivated in a YPD medium containing 15% glucose. However, the tendency toward piezotolerance of the strain cultivated on *koji* extract medium with the high glucose concentration may coincide with the increased piezotolerance for increasing initial glucose concentrations in YPD media.

### 3.4. Adenosine Triphosphate (ATP) Concentrations in Yeast Cells Cultivated in a YPD Medium Containing 2% or 15% Glucose Using QUEEN Protein

**Figure 6:**
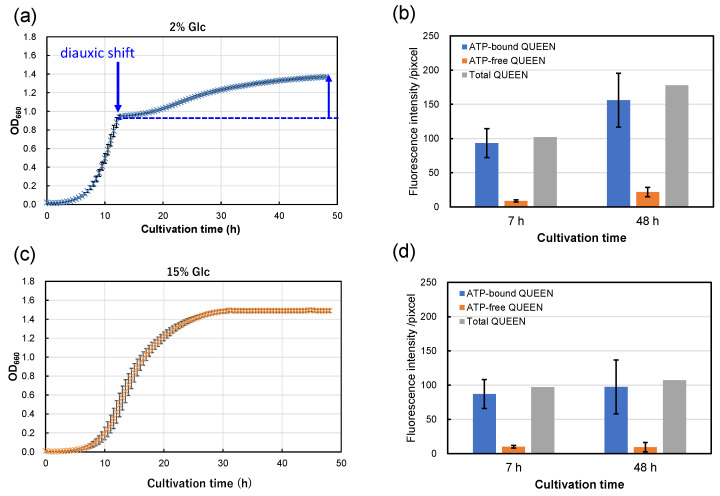
Growth curves of strain MTY3143 in a YPD media containing 2% (*w*/*v*) (**a**) and 15% (*w*/*v*) (**c**) of glucose at 30 °C. Average values from eight assays are shown with error bars, indicating standard deviations. The fluorescence intensities of ATP-bound (blue boxes) and ATP-free QUEEN proteins (orange boxes) at 7 h and 48 h cultivation in a YPD media containing 2% (*w*/*v*) (**b**) and 15% (*w*/*v*) (**d**) of glucose at 30 °C. The data represent averages of 25 randomly selected cells, with error bars indicating standard deviations. Sums of the fluorescence intensities of ATP-bound and ATP-free QUEEN are also shown in gray boxes.

From the results above, strain S9arg shows increasing piezosensitivity with increasing glucose concentrations in YPD media. The cultivation periods after diauxic shift shorten with increased glucose concentrations in YPD media, being approximately 40 h (2% Glc), 38 h (5% Glc), 35 h (10% Glc), and 0 h (15% Glc; apparent diauxic shift was not observed). Since yeast mutant strains deficient in aerobic respiration show increased piezosensitivity, the aerobic respiration function after diauxic shift may possibly be important for piezotolerance. Thus, we carried out an evaluation of intracellular ATP levels in single cells using strain MTY3143 [19], which is the genetically encoded fluorescent ATP biosensor QUEEN [21]. Strain MTY3143 was cultivated in a YPD media containing 2% glucose and 15% glucose at 30 °C for 48 h (Figure 6a,c). When cultivated in a YPD medium containing 2% glucose, the strain showed exponential growth for 12 h, and then a diauxic shift was observed. Subsequently, growth decelerated, with slower secondary growth to 48 h (Figure 6a). In contrast, the strain did not show an apparent diauxic shift when cultivated in a YPD medium containing 15% YPD medium (Figure 6c). The growth characteristics of strain MTY3143 were similar to those of strain S9arg.

Following cultivation, the cells were subjected to epifluorescence microscopic observation. The fluorescence signals of ATP-bound and ATP-free QUEEN proteins were approximately comparable after 7 h cultivation (in the exponential growth phase), regardless of the glucose concentration in the YPD medium (Figure 6b,d). However, after 48 h of cultivation, the fluorescence signal of ATP-bound QUEEN proteins showed a tendency to be higher in the cells cultivated in a YPD medium containing 2% glucose than that cultivated in a YPD medium containing 15% glucose (Figure 6b,d). The results suggest that higher levels of intracellular ATP were accumulated by cultivation in a YPD medium containing 2% glucose than by cultivation in a YPD medium containing 15% glucose. The fluorescence signal of ATP-free QUEEN proteins was higher in the cells cultivated in a YPD medium containing 2% glucose than that cultivated in a YPD medium containing 15% glucose. The results indicate that intercellular metabolism for protein synthesis is more active after cultivation in a YPD medium containing 2% glucose than that cultivated in a YPD medium containing 15% glucose.

Since strains S9arg and MTY3143 have aerobic respiratory ability and similar growth characteristics, the energy status of strain S9arg was suggested to be similar to that of strain MTY3143. The energy status of strain S9arg after 48 h cultivation in a YPD medium containing 2% glucose would be relatively high based on high ATP levels. The cells of strain S9arg could recover from damage caused by HHP and exhibit high piezotolerance. When the strain was cultivated in a YPD medium containing 15% glucose, the cells generated energy only via anaerobic ethanol fermentation throughout 48 h of cultivation. Glucose in the YPD medium would be consumed and used up after around 16 h; subsequently, the strain had to maintain its cells under limited energy. The energy status of strain S9arg after 48 h cultivation in a YPD medium containing 15% glucose would be relatively low based on low ATP levels. The cell damage caused by HHP could not sufficiently be repaired, resulting in decreased piezotolerance.

### 3.5. ATP Concentrations in UV1 Cells Cultivated in a YPD Medium Containing 2% or 15% Glucose

**Figure 7:**
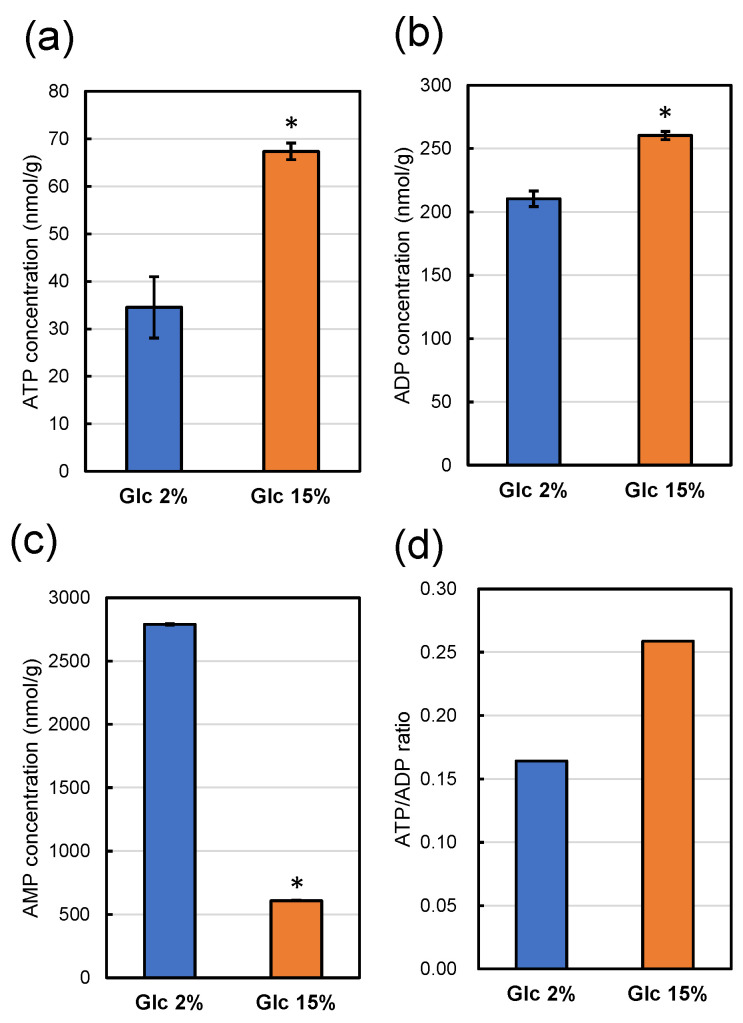
ATP (**a**), ADP (**b**), and AMP (**c**) concentrations in 1 g of dry weight of strain UV1 after cultivation for 48 h at 30 °C in a YPD medium containing 2% (*w*/*v*) (blue boxes) and 15% (*w*/*v*) (orange boxes) of glucose. All values represent averages of three measurements, with error bars indicating standard deviations. Significant differences (*p* < 0.05) of the concentrations with those on YPD medium containing 2% (*w*/*v*) glucose are indicated with asterisks. The ATP/ADP ratios are shown in panel (**d**).

Since strain MTY3143 has intact aerobic respiratory ability, the strain could not be used to analyze intracellular ATP levels of respiratory-deficient mutant strain UV1. We analyzed intracellular ATP, ADP, and AMP of strain UV1 using CE-MS after cultivation in a YPD media containing 2% glucose and 15% glucose at 30 °C for 48 h (Figure 7).

The intracellular ATP concentration of strain UV1 cultivated in a YPD medium containing 15% glucose was significantly higher than that cultivated in a YPD medium containing 2% glucose (Figure 7a). The intracellular adenosine diphosphate (ADP) concentration of strain UV1 cultivated in a YPD medium containing 15% glucose was also significantly higher than that cultivated in a YPD medium containing 2% glucose (Figure 7b). The ATP/ADP ratio for strain UV1 cultivated in a YPD medium containing 15% glucose was higher than that cultivated in a YPD medium containing 2% glucose (Figure 7d). In contrast, the intracellular adenosine monophosphate (AMP) concentration of strain UV1 cultivated in a YPD medium containing 15% glucose was remarkably lower than that cultivated in a YPD medium containing 2% glucose (Figure 7c). These results indicate that strain UV1 cultivated in a YPD medium containing 2% glucose has a relatively poorer energy status than that cultivated in a YPD medium containing 15% glucose: a lower ATP concentration, as well as a lower ATP/ADP ratio and higher AMP concentration.

In this study, we chose two types of methods for evaluating intracellular ATP concentrations: fluorescence analysis using the QUEEN strain (Section 3.3) and measurement using CE-MS (Section 3.5). The advantage of the former method would be high sensitivity, which can analyze ATP concentration in individual cells, whereas the disadvantage would be that ADP and AMP concentrations could not be measured. In contrast, the latter method has the advantage that ADP and AMP concentrations cannot be measured, whereas it has the disadvantage of limited sensitivity. The combination of these two methods would be important for precise evaluation of the intracellular ATP concentration.

Since the mutant strain UV1 is genetically deficient in aerobic respiration, the cells would generate energy via anaerobic ethanol fermentation throughout 48 h of cultivation in a YPD medium containing 2% glucose. Glucose in the YPD medium would be consumed and used up after around 10 h; subsequently, the strain maintained its cells under limited energy. The energy status based on lower ATP levels after 48 h of cultivation would be relatively low so that cell damage by HHP could not sufficiently be repaired, resulting in piezosensitivity. When the strain was cultivated in a YPD medium containing 15% glucose, the cells generated energy only via anaerobic ethanol fermentation throughout 48 h of cultivation, similar to that cultivated in a YPD medium containing 2% glucose. However, due to the high initial glucose concentration, glucose in the YPD medium was consumed and used up after a longer cultivation period than the culture in a YPD medium containing 2% glucose, by around 16 h. Even after 48 h of cultivation for cell maintenance, the energy status based on higher ATP levels was relatively high so that cell damage by HHP could be repaired, resulting in increased piezotolerance.

Numerous important studies have been conducted concerning factors affecting microbial inactivation under HHP [8,15]. The HHP conditions, consisting of the pressure levels and HHP treatment time and temperature, primarily affect the inactivation of microorganisms, including budding yeast [12,13]. In addition to these HHP conditions, the inactivation of microorganisms also depends on extracellular environments such as the suspension media composition or food, including sugars and ionic compounds with their concentrations [22], pH, and water activity [8,15]. The types of microorganisms also affect their inactivation. Our piezosensitive mutant strains derived from *S. cerevisiae* demonstrated genetic background effects from HHP inactivation. The growth phase has also been demonstrated to be an important factor affecting HHP inactivation. Bacterial cells in stationary phases are reportedly less susceptible than exponential-phase cells to environmental stresses, including HHP.

Our study demonstrated that the intracellular energy status, evaluated as ATP concentration, shows a correlation with piezotolerance or piezosensitivity in *sake* yeast strains. The energy status is affected by numerous intracellular and extracellular conditions. A possible mechanism may be that the conditions affect the intracellular energy status, which decides the piezotolerance or piezosensitivity of microorganisms under HHP. Recently, there have been several reports that HHP may injure a proportion of microorganisms, where the repairing ability of injured cells affects the piezotolerance or piezosensitivity of the microorganisms [15,23]. We previously reported the importance of cell damage causing growth delay for high-pressure inactivation of *S. cerevisiae* [24]. Hao et al. demonstrated that the intracellular ATP concentration showed an upward trend after repairing *Escherichia coli* strain O157:H7 cells that were sub-lethally injured by HHP [25]. Our present results show that higher intracellular energy status resulted in increased piezotolerance of the S9arg parent strain as well as the UV1 mutant strain, which could be explained by the greater repairing ability of HHP-injured cells. The mechanism for increased piezotolerance through increased intercellular energy states may be useful against other environmental stresses.

In this study, an increased piezosensitivity of strain S9arg after laboratory-scale *sake* brewing test was observed compared with that cultivated in a YPD medium containing 2% glucose. This result may account for the possibly low energy status of the strain after the brewing process due to a high concentration of glucose during the early days of brewing, which was similar to cultivating in a YPD media containing 10 to 15% glucose. A low aeration efficiency of the brewing process compared with that in shaking cultivation would restrict the aerobic respiration of strain S9arg. Other factors during the brewing process, such as lower temperature, may also additively increase piezosensitivity. In contrast, the UV1 and NM1 mutant strains, which were genetically deficient in aerobic respiration, did not show apparent alterations in piezotolerance or piezosensitivity. The glucose concentration during the brewing process would be similar to cultivating in a YPD media containing 10 to 15% glucose, suggesting increased piezotolerance. However, other factors during the brewing process, such as lower temperature, may oppositely increase piezosensitivity, resulting in no apparent alterations in piezotolerance or piezosensitivity.

## 4. Conclusions

In this study, we investigated the HHP inactivation behaviors of Niigata *sake* yeast strain S9arg and its aerobic-respiratory-deficient mutant strains due to the deletion of genes for the respiratory chain in mitochondrial DNA after cultivation in a YPD media containing 2% to 15% glucose, as well as in *moromi* mash in a laboratory-scale *sake* brewing process. The piezotolerance of strain S9arg, shown after cultivation in a YPD medium containing 2% glucose, decreased to become piezosensitive with increasing glucose concentrations in YPD media. In contrast, the piezosensitivity of a mutant strain UV1, shown after cultivation in a YPD media containing 2% glucose, decreased to become piezotolerant with increasing glucose concentrations in YPD media.

The intracellular ATP concentrations were analyzed for an *S. cerevisiae* strain MTY3143 with intact aerobic respiratory ability, as well as the UV1 strain. The higher concentration of ATP after cultivation suggests a higher energy status, which is closely related to higher piezotolerance for yeast strains. A higher energy status could repair injured cells caused by HHP treatment, resulting in increased piezotolerance. This proposed mechanism could explain the increased piezosensitivity observed in strain S9arg after laboratory-scale *sake* brewing, compared with that cultivated in a YPD medium containing 2% glucose. The conditions of *moromi* mash during the *sake* brewing process resemble those for cultivating in a YPD media containing 10 to 15% glucose. The low aeration efficiency of the brewing process compared with that under shaking cultivation restricts the aerobic respiration of strain S9arg.

The intracellular ATP concentrations of strain S9arg were not provided in the present study. The assays are used to measure the intracellular ATP concentration using several *S. cerevisiae* strains with intact mitochondrial functions including strain S9arg, as well as strains with deficient mitochondrial functions. We would provide results sufficient to draw the general mechanism of increased piezosensitivity as a future study. Other factors during the brewing process, such as lower temperature, may also additively increase piezosensitivity; such factors should also be investigated.

To our knowledge, this is the first report demonstrating the relationship between variation in piezotolerance or piezosensitivity and the intracellular energy status of *S. cerevisiae*. The proposed mechanism may be useful against other environmental stresses. This study may be useful for applying HHP processing to inactivate microorganisms by better understanding the variations in piezotolerance and/or piezosensitivity caused by cultivation conditions and/or genetic factors. A better understanding of the HHP inactivation of yeast strains could provide optimization of HHP processing conditions. The influence of piezosensitive strains on the quality and shelf-life of *sake* processed would also be important for HHP application for *sake* manufacturing.

## Figures and Tables

**Table 1:** Inactivation rate constants (*k*) of yeast strains S9arg and UV1 under HHP treatment at 200 MPa at ambient temperature (approximately 20 °C) after cultivation for 48 h in a YPD media containing 2% (*w*/*v*) to 15% (*w*/*v*) of glucose.

	S9arg		UV1	
Glucose Concentration	Diauxic Shift	*k* (s^−1^)	Diauxic Shift	*k* (s^−1^)
2% (*w*/*v*)	+	0.0013 ± 0.0007	−	0.0221 ± 0.0016
5% (*w*/*v*)	+	0.0034 ± 0.0025	−	0.0123 ± 0.0027
10% (*w*/*v*)	+	0.0058 ± 0.0007	−	0.0148 ± 0.0008
15% (*w*/*v*)	−	0.0086 ± 0.0023	−	0.0129 ± 0.0017

*k* values represent averages from three assays, with standard deviations, with one exception: the 10% glucose results show averages of two assays. +, diauxic shift observed; −, diauxic shift not observed.

## Data Availability

The original contributions presented in the study are included in the article, further inquiries can be directed to the corresponding author.
